# Love and Romantic Relationships in the Voices of Patients Who Experience Psychosis: An Interpretive Phenomenological Analysis

**DOI:** 10.3389/fpsyg.2020.570928

**Published:** 2020-10-26

**Authors:** Magdalena Daria Budziszewska, Małgorzata Babiuch-Hall, Katarzyna Wielebska

**Affiliations:** Department of Psychology, University of Warsaw, Warsaw, Poland

**Keywords:** love, romantic (love), schizophrenia, psychosis, qualitative study, interpretative phenomenological analysis, rehabilitation, illness

## Abstract

Love is a universal experience that most people desire. A serious, long-term, and stigmatized illness makes entering and maintaining close relationships difficult, however. Ten persons, who have been diagnosed with schizophrenia, and lived with their illness for between years and decades of their lifetimes, shared their stories. They reported how the illness has influenced their emotional experiences regarding love and their intimate relationship experiences. We present here a qualitative interpretative phenomenological analysis (IPA) of their narratives. This analysis has been done with an explicit intention to give voice to the patient’s perspective. The results highlight how illness adjustment and hospitalizations have an alienating effect on relationships through stigmatization and self-stigmatization; how illness creates psychological obstacles to love, such as diminished trust toward oneself and others; and how long-term patients experience practical difficulties in creating and sustaining relationships, such as poverty. Moreover, we show how patients experience changes in sexuality and the risks involved in it and discuss possible coping strategies from their perspectives.

## Introduction

Love is a universal experience that most people desire. A serious, long-term illness, like schizophrenia, introduces a set of specific obstacles for entering and maintaining close relationships, however. To tell the story of love, and what happens to it, when someone is also burdened with psychosis, is an exercise that requires a great deal of courage. Ten patients, who have been diagnosed with schizophrenia, and lived with their illness for a period of between years and decades of their lifetimes, shared their stories. They reported how the illness has influenced their emotional experiences and life trajectories. We present a qualitative interpretative phenomenological analysis (IPA) of their narratives. This analysis has been done with an explicit intention to give voice to the patient’s perspective. Presenting this perspective in a systematic way can help medical professionals to understand the lived experiences and the context of the illness. Love, through the creation of meaningful relationships, stable support networks, community participation patterns, and life goals is a central, yet often overlooked, aspect to the trajectories of illness and recovery.

Love is one of the universal themes of humankind, reflected in many cultural narratives and personal stories. In the context of romantic relationships, finding a partner and starting a family is one of the common goals for young people and a developmental milestone in the course of life (Redmond et al., 2010). Sternebrg (2006) proposed a general integrative theory of love, claiming that, in addition to the elements of intimacy, passion, and commitment, love can be understood as a story in itself. He stresses that the narratives people tell and believe—what is love, both love generally and love in their personal lives—shape the experiences that they have in this area. Therefore, listening to personal narratives about love is a privileged way to inquire into this topic.

Love is more than just passion, sexual desire, and erotic fascination expressed in romantic relationships. Apart from erotic feeling (*eros*) as an emanation of human sexuality, love can also be viewed as an emanation of human spirituality (*philia*, *storge*, *agape*, and *thelema*) expressed within friendship, brotherhood, and parent-and-child relationships, or as a spiritual attitude toward a broader environment or God (Davidson, 2011; McCarthy-Jones and Davidson, 2013). Both high and popular culture and listening to people’s experience demonstrate that love is at the depth of human existence, a central thread in the fabric of life. McCarthy-Jones and Davidson (2013) describe love as central to the onset and the immersion into psychosis, when the love of close ones and affirmation by society is often lost, and central in the process of recovery. In the context of voice-hearing experiences, they stress that those experiences are common in many cultures for those who are extremely isolated or who have lost loved ones.

The desire for love, meaningful personal relationships, romance, and family is well documented in persons with schizophrenia (Davidson and Stayner, 1997; Redmond et al., 2010; Davidson, 2011), as well as present in the clinical experience. On the other hand, problems like social isolation, loss of romantic interest, and difficulties in creating and maintaining interpersonal relationships are diagnostic hallmarks of schizophrenia and contribute to a worse prognosis (Hooley, 2010). Deficits in social cognition especially influence poor social functioning (Penn et al., 2008; Savla et al., 2012). This might give the impression that love and schizophrenia are incompatible, and this opinion reflects a popular stereotype about the illness and could be part of self-stereotyping in patients (Redmond et al., 2010).

In an extensive qualitative study, Davidson (2003) explains that the clinical perspective shared by medical professionals on persons with schizophrenia may too frequently be described by the metaphor of an “empty shell,” where there is not much to address, such that needs relating to love may remain unnoticed. In contrast to that, he suggests that patients’ families often understand their family members with the metaphor of a poker player hiding his or her emotions. However, patients’ first-person accounts of emotional life and sense of the self suggest what he calls a “caged panther” metaphor as the metaphor of the self, relating to the poem by Rilke (1981). There is a sense of immobility and stifled desire, of a self that is capable of feeling and desirous of relationships and emotions (Davidson, 2003), but which is somehow captured by the illness, to the point that it cannot express the full extent of its potential.

### Schizophrenia and Discrimination in the Area of Relationships

“Bottom of the totem pole, very lowest of the low, scum of the earth”—this is how one of the participants with the experience of psychosis in a study by Rice (2008) described her position in society. Indeed, individuals with schizophrenia are among the most stigmatized groups. In international studies (Thornicroft et al., 2009) done in over 27 countries, levels of experienced and anticipated discrimination of persons with schizophrenia rated by themselves were high in all domains. Notably, 27% of respondents with schizophrenia experienced discrimination in romantic relationships and sexual relationships, and 55% anticipated this kind of discrimination. A serious problem lies also in the self-stigmatization of this group, which leads to low self-esteem and isolation of patients. In a GAMAIN–Europe study done in 14 European countries, almost half of the participants (41.7%) reported moderate or high levels of self-stigma (Brohan et al., 2010), and this was predicted, inter alia, by poor social contact. Schulze and Angermeyer (2003), in a study using a focus-group methodology, have shown that all three groups—patients, their families, and mental health professionals—identified personal relationships as the biggest area where the stigma of mental illness is present. Discrimination and stigma are also reported in contacts with medical staff. Results of a study by Thornicroft et al. (2009) show that 38% of participants felt disrespected by mental health staff, with higher ratings in post-communist countries, where, for example, 23.4% felt strongly disadvantaged if they chose to consult medical staff about starting a family. In a Polish study by Cechnicki et al. (2007), 87% of over 200 psychiatric patients diagnosed with schizophrenia or schizophrenia spectrum disorder have experienced interpersonal rejection, 50% the loss of contact with someone close. Two-thirds of the participants with this diagnosis believed that others do not want to have a partner with this diagnosis. Indeed, schizophrenia is connected to a lower likelihood of getting married, especially for men, and to higher chances of broken marriages for women. An existing marriage before the first onset is, however, a highly predictive measure of pre-illness adjustment and a factor in better future outcomes (Thara and Srinivasan, 1997).

### Present Study and Rationale for Using the IPA

The objective of the interpretative phenomenological analysis (IPA) is to gather a rich and in-depth understanding of how individuals experience a particular phenomenon, and then to understand and interpret this experience as it is shared within a small group of participants (Pietkiewicz and Smith, 2014). In the present study, we would like to understand how patients, diagnosed with schizophrenia and with the experience of psychosis, approach and give meaning to love, especially in the context of romantic relationships. IPA employs principles of a hermeneutic focus on the individual experiential world and phenomenological lenses to see something from within (Larkin et al., 2006). We believe that love, as a very intimate topic, connected to personal meanings, a sense of purpose, and identity, can be approached via the IPA method with due respect to the individuals who have shared their experiences. Moreover, the IPA serves the additional purpose of “giving voice and making sense” of the experience, such that it helps to understand the inner world of other people (Larkin et al., 2006). Such understanding can be very important in the area of healthcare, important for society for the prevention of discrimination and stigma and for mental health professionals to understand their patients.

Redmond et al. (2010) conducted one of the few interpretative phenomenological studies about the significance that young people with psychosis ascribed to love and romantic relationships. They found that young patients associated love with normality and recovery, wished for it, but also perceived love and psychosis as incompatible with each other, and romantic relationships as more burdened by risk for them. Hope and various attempts to approach relationships slowly and in a safe way were characteristic for this young group. However, no studies about the perspectives of other then the young, first-onset-group patients have been conducted. Our study aims to fill that gap. Older and long-term patients who have more life experience can speak about how romantic relationships have proceeded in their lives over many years, and from a different time perspective.

## Materials and Methods

### Participants

The participants in this study were 5 women and 5 men, each of whom had experienced severe mental illness and had a medical diagnosis of schizophrenia. At the time of the interview, six of them were patients in an outpatient psychiatric clinic in Warsaw, which represents an urban mental health institution within a public health system. One participant was a member of a patients’ club at the clinic, and another was a member of a support group for individuals with mental illnesses associated with the clinic. The last two participants were members of social networks comprised of prior participants of this study. All participants were under ongoing care of psychological and psychiatric professionals and received medical and, in some cases, psychological treatment. No participant was in the acute stage of his or her illness at the time of the study, but all had earlier in-patient experiences. The mean age of participants was 34.4 (*SD* = 6.07). Three persons had higher education, five had a secondary education, and two had vocational education. None of the participants were employed at the time of the interview. Eight participants lived with their families, one lived in a patients’ hostel run by the clinic, and one lived alone. All participants were heterosexual and none had children. Names have been changed, and all identifying details have been avoided in quotations to ensure confidentiality.

### Interview Protocol

We used a semi-structured interview protocol, involving introductory general questions about understanding love, questions about life and emotional experiences in relationships, hopes, and the perceived impact of illness on relationships. This protocol was then employed as in-depth interview, in accordance with the IPA method (Pietkiewicz and Smith, 2014).

### Data Collection and Ethical Standards

A major outpatient psychiatric clinic in Poland allowed for the participants’ recruitment. The inclusion criteria were as follows: being diagnosed with schizophrenia and having an experience of psychosis, and not being in an acute phase of illness. The research project and interview protocol received the institutional approval of the ethical committee prior to the study. Ethical guidelines followed were consistent with American Psychological Association research ethics recommendations (all authors are psychologists) and included informed consent, including publication of social and demographic characteristics of the study group and using direct quotes from the participants; the right to withdraw from the study at any moment; recording; and recorded data safety regulations. [KW] conducted all interviews personally, choosing the time and place at the patients’ convenience. All interviews were tape-recorded and transcribed verbatim. These lasted between 40 min and 1 h 35 min.

### Data Analysis and Reliability

After a familiarization phase, and discussion between authors, who were first making preliminary notes and comparing and discussing their insights, the first author performed a systematic analysis. She started with notes and preliminary emerging themes and then refined and corrected initial insights through an iterative analytic process, to create a set of subthemes and main themes to higher levels of abstraction. Additionally, this set of themes was applied to the text and checked line by line against the transcripts in order to evaluate how well they reflect the material. The analysis was subsequently reviewed by two other authors and developed further, as their insights were incorporated into the analysis. This process resulted—after a few review and discussion phases—in all authors’ agreement about the final analysis, as well as the final written report. The procedure was similar to that described by Pietkiewicz and Smith (2014). As an additional way of ensuring reliability, results were presented and discussed at a conference on qualitative methods, with researchers who specialize both in the IPA methodology and in the topic of psychosis.

#### Strategies of Ensuring Reliability

The following concerns regarding qualitative research transparency and quality were taken into account. First, we guided all study procedures in line with the study’s goals, which concern understanding individual experiences. Second, we take a phenomenological perspective as epistemology and use interpretative phenomenological analysis. Thus, during the text analysis, we focus more on participants’ subjective perspective and the meanings attached to the experiences than on factual content. Consequently, we paid attention to each participant’s experiential truth and were aware of the way in which it was communicated in relationship to the listener. We avoided over-generalizing similarities and purposely paid attention to the individual aspect of experiences. Moreover, we report the result in a personalized manner, linking stories to persons and giving voice to the participants. The analysis was done in a systematic way and reviewed both inside and outside the research team. We engaged in self-reflexivity and report this in the discussion section. A quality criterion in our type of research must be relevant for practitioners, so we aimed at communicating our results in a manner to be transferable and applicable in different contexts. The general principles regarding quality are similar to the general overview proposed by Meyrick (2006).

## Results

Five salient themes were created from the interviews. Table 1 presents an overview.

**TABLE 1:** Master themes and subthemes in interviews.

	**Master themes**	**Subthemes**
(1)	Illness adjustment is all-consuming at first and can lead to isolation	Identity work and adjustment
		Impact of first hospitalizations

(2)	Inner obstacles: illness-induced changes in experiencing love can be obstacles to entering and maintaining romantic relationships	Intimacy and emotions
		Inner chaos, disorientation, difficulties in understanding others’ intentions
		Suspicions and distrust

(3)	External obstacles: low status, discrimination, and poverty make it difficult to establish and maintain close relationships	Low status and discrimination
		Poverty
		Physical appearance

(4)	Experiencing sexuality becomes more challenging	Lowered trust in self and others
		Women’s experiences: risk of sexual abuse and need for stable relationships
		Men’s experiences: romantic ideals and competition
		Voluntary sexual abstinence

(5)	Ways of coping	Love is understood as a universal value
		Other objects of love
		Loneliness
		Expectations for the future

### Theme 1: Illness Adjustment Is All-Consuming at First and Can Lead to Isolation

In the participants’ memory, the theme of illness onset stands out, together with the need to adjust to new life situations and the impact of first hospitalizations. The onset of schizophrenia was a cutoff point of their narratives, separating their lives into “before” and “after” landmarks. It was related not only to their personal lives but also to their histories of love and romantic relationships.

#### Identity Work and Adjustment

For many participants, the experience of psychosis resulted in the need for a redefinition of the self-image and their image of the world. Alice, a woman in her late twenties, notices and expresses the change in herself and the resulting need for identity work the following way: *I seem to be completely new to myself in many ways.* After illness onset, she feels she has to learn anew who she is. This renewed identity work is intense and does not leave very much room for relationships. Harry, who is in his forties and was diagnosed 4 years ago, expressed it by saying:

The beginning of this illness is that you don’t know what you want, where you stand, and who you are. You have millions of questions in your head so you just forget about stuff like love. It’s set on the back burner, right? Only after a certain period of time, when you pull yourself together, you start thinking about stuff like that, right? The first stage is ME, only.

First-time illness experiences seem to pull patients out of the world of interpersonal relationships and drag them into loneliness.

#### Impact of First Hospitalizations

In addition to the need to adapt to the illness, the patients’ narratives exhibited another theme—the theme of the first hospitalization. They name it as a factor that most strongly pulls them out of their contemporary lives, including their relationships, and most strongly stigmatizes them. It also can provoke self-stigmatization. James, a young man in his early twenties, stated: *“I had this method where I (…) [while in the hospital] cut myself off from everyone. I just didn’t want anyone seeing me in this state. My friends knew about it, but I just didn’t want anyone to see me.”*

James was left by his long-term girlfriend after he was released from the psychiatric ward.

My partner was a girl younger than me. She left me when I was in…after I got out of the hospital. I think my illness terrified her to some extent. Meanwhile, she said this phrase, that…when we were separating, she said that she has no intention of crying due to my illness. (…) Maybe she was scared of the future that didn’t look so rosy. Being with someone suffering from an illness like that.

Several participants experienced the severing of both romantic relationships and friendships during the first hospitalization, or some time later. Either the patients were isolating themselves from their loved ones as the psychosis progressed or their partners were leaving some time after the onset, scared off by the illness and its consequences.

### Theme 2: Inner Obstacles: Illness-Induced Changes in Experiencing Love Can Be Obstacles to Entering and Maintaining Romantic Relationships

Psychosis entails experiences unknown or alien to other people. The second theme in the participants’ narrations was the theme of the consequences of such experiences on the romantic relationships.

#### Intimacy and Emotions

The influence of positive symptoms (for example hearing voices) on the world of thoughts and emotions is an intense and personal experience. Delusions of influence and control (I’m controlling things or being controlled by someone), the idea that others could hear my thoughts and I could hear theirs, share a common theme of blurring the psychological boundaries, the distinction between oneself and the external world or another person. In consequence, romantic relationships may be experienced as a sort of fusion, which can at once be both alluring and unsettling. Alice describes this:

My ideal…well, right now, it’s a problem because I’m after psychosis, really severe psychosis, where I was hearing voices all day, non-stop. For me that’s a very intimate form of bond. If you hear someone’s voice in your head, and that voice is with you non-stop, then it’s like no one else can become closer to you than that voice in your head. So in a way, this feeling has remained, that the greatest form of intimacy I ever had was through the illness, and I’ll never have this rapport with anyone like that flow of thoughts in my head, like a connection of thoughts. (…) So, for me, the perfect guy would be a guy that could read my mind and could influence my thoughts, something like in “Twilight”.

The desire for intimacy and closeness is a deep-seated need. For Alice, the intense experiences associated with psychosis stand in stark contrast to her normal life and the relationships in it, relationships that do not have the same immediacy and intensity.

However, not all patients experience their symptoms as something to be shared with their partners—the unusual character of the experiences can also lead to secrecy. The intimacy of the patients’ relationships to their symptoms, the personal nature of the symptoms, and the distrust that comes with certain forms of schizophrenia can all preclude any communication on this topic within a relationship. John mentioned when talking about his former partner: *“I didn’t touch that topic. She knew I was ill. But I left that for myself, my feelings, and my experiences during the illness.”*

The theme of experiencing closeness with another person co-occurs with the theme of a perceptible change in experiencing emotions. Alice, again: “*The thing is, right now my emotions are sort of extinguished, they’re the opposite of vibrant. (…) It’s just that some things don’t reach me, or they bounce off.”* After having experienced psychosis, Alice sees herself as muted, unable to feel as intensely. She relates her unreadiness—for initiating romantic relationships, for falling in love—to her difficulties in experiencing things. “*Because if you’re falling in love, those emotions have to be there. They just have to …”*

#### Inner Chaos, Disorientation, and Difficulties in Understanding Others’ Intentions

Chaos and disorientation often accompany psychosis. For example, in an interview with Emily, a woman in her forties, the sense of disorientation and chaos dominates the conversation. Her love story begins like: *“There was a man, who … I was then after some ordeals, big psychological ones …I was searching for some (…) comfort, I wanted to be accepted more.”* Later in the interview, within a single utterance, she moves from great tenderness toward this man to sharp accusations of abuse, from disjointed fragments of memories to elaborate interpretations of his behaviors.

I thought about him some, right? But at that moment, when I felt that he had awakened me emotionally, that I can’t deal with it. I dealt with that somehow, somehow alone, but … I felt … and then he called me … he asked if I’ll come out, texting me “Come out!” “Come!” with exclamation marks and all. (…) At some moment, I felt that it’s … maybe there’s something between us, but then, after these experiences, I felt that he’s just lonely.

Within her own love story, rich in hedges, question marks, and linguistic expressions of uncertainty, she talks about guessing her lover’s intentions rather than knowing them. Emily’s long story about love is missing many important details. The listener does not know what happened and when. She, herself, is also confused about the actual meaning of her story. Other patients’ narratives also highlighted that the effort put into making sense of the fragmented experience, including sexual experience, can be remarkable.

#### Suspicions and Distrust

Interpreting one’s own experiences during psychosis is made even harder by the fact that positive symptoms frequently inject unsettling and threatening inputs that are difficult to assimilate and which inundate the person with images of aggression and violence. Eva, a woman in her mid-thirties, has been ill for all of her adult life. In her interview, there were frequent motifs of rape, violence, and sexual abuse that, in her mind, were constantly happening to many people around her. She assigns such sequences of events to the man she loves, saying: *“In high school, he was a victim of bullying, he was beaten, raped, and robbed. In general, he was persecuted because he’s a minority. He was also …I mean, his mother was raped too (…).”*

In her life story and that of all who are close to her, the motif of rape appears dozens of times. It is difficult to think of interpersonal relations as a source of support and satisfaction when such threatening thoughts overflow the consciousness. The blending of experiences and symptoms, along with the frequent paranoid component of the illness, can result in the emergence of deep mistrust. Suspicions, anxiety, and fear can affect the patient’s attitude toward love. Negative expectations often characterized the participants’ narratives.

### Theme 3: External Obstacles: Low Status, Discrimination, and Poverty Make It Difficult to Establish and Maintain Close Relationships

Internal obstacles are not the only challenges faced by the study participants. The external reality of living with an illness can be just as challenging.

#### Low Status and Discrimination

Persons with long-term mental illness commonly experience social stigma, along with self-stigmatization. They are not seen, nor do they see themselves as attractive partners in romantic relations. Martha, a middle-aged woman with a nearly 20-year history of being ill, describes recurring experiences of romantic rejection due to the illness. Her relationships were often short-term, and primarily sexual. Despite her desire for a long-term relationship, the men always left her. Even though her consecutive partners were rather unattractive men—described, among others, as an alcoholic met on the street, and a penniless drug addict who wanted to use her for her disability benefits—her status as a mental health patient inevitably resulted in the termination of the relationship.

That alcoholic… I was just sitting on a park bench. He came up to me. We started talking. Later he invited me to his place, we had sex, but … he said he doesn’t want to end it… he doesn’t want to just use me… He was a good guy. Unfortunately, he found out I’m in [name of major psychiatric hospital] and he didn’t want to be with me anymore.

The study participants often expressed an understanding that the illness can pose a great challenge for potential partners but that understanding does not make the rejection any less painful. Informing potential partners about the illness was an important issue, where the problem was in choosing an appropriate moment to confess to it. The perceived risk of rejection is very high.

The theme of the low status of mental health patients, especially those suffering from psychosis, is angrily, but also with sharp irony, described by Jack, the longest-suffering of all of the male participants. He expresses his pride and dignity—despite hardships and deficiencies—through distancing himself from society and openly expressing his anger at the social status of the mentally ill. For example, when asked where he would take a girl on a date, he says: *“To the dump. – Interviewer: Where? What do you mean? – (R): The landfill (laughing)…. – (I): But wouldn’t she run away from the landfill? – (R): C’mon, I’m kidding.”* His joke reveals something about his inner experience—he chooses the proverbial landfill, a place associated with the very bottom of the social pile and with outcasts, as a setting for love for someone in his situation. His responses, despite frequent verbal self-stigmatization (“us mentals”), bear the hallmarks of personal dignity through their simultaneous expression of anger and irony. He clearly made his point that, in his experience, a mentally ill man cannot expect much from women.

#### Poverty

Stigmatization, self-stigmatization, unattractiveness, and rejection in romantic relations are caused not only by the illness itself but, according to a few participants, are also greatly amplified through poverty—a consequence of the illness. Poverty makes any romantic relationship difficult. Eva describes an early relationship:

That relationship didn’t survive because we didn’t have, I didn’t have money. (…) That’s why I knew that after high school, I’ll probably go on disability. I had no way of supporting myself, no work opportunity, and he didn’t have work opportunities either, (…) The problems were mainly financial.

Currently, the frequency of Eva’s social outings and interactions—both romantic with her special friend and non-romantic with her other friends—is determined by the contents of her wallet. She self-identifies with poverty, for example she believes that most men see her primarily and first as a poor person, and this makes her an unattractive partner, even if the theme of illness does not come up. After years of illness, it is the poverty, not the illness, that consumes a lot of space in her everyday stories and has the greatest impact on her day-to-day life and her romantic possibilities.

What Roman thinks is that his situation would be much better if he could work: “*I’d like to go to work, sure, but nobody wants me.”* In his narration, work is linked with love. Roman is afraid that if he were to date a woman and she wanted him to buy her a little something, he would not be able to afford it, and that would undermine his role as a man, reinforcing the stigma. Participants often see financial resources as a deterrent to attractiveness and independence in establishing and maintaining relationships. Poverty adds to the illness and becomes a determinant of multiple, cross-sectional stigma.

#### Physical Appearance

Medication-related weight gain is a problem for most of the men and women who suffer from this negative consequence, as it adds to their stigmatization in a particularly painful way. Obese individuals in Western culture are viewed as romantically unattractive, and if someone is ill and poor, and also obese, their social status is critically low. Such individuals can be met with disdain and be the target of hate speech. Among the women, Eva, who was slender as a teenager and later gained a lot of weight due to many years of pharmacotherapy, most clearly and bitterly expressed the motif of physical attractiveness. She speaks about her romantic friendship: “*As far as the ideal woman, I for sure am not his ideal, and he isn’t mine.”* Love or friendship can be sustained even when the individuals are imperfect, but the common cultural patterns of attractiveness, which put a strong emphasis on physical beauty, are unforgiving for Eva. She recalls many conversations with men involving this topic. In effect, she is afraid of rejection and knows it well: “*I’m afraid of rejection, that if I were to get emotionally involved, like it’s been many times before when I got involved with this or other guy and later got rejected…It was a very sad emotional state.”*

### Theme 4: Experiencing Sexuality Becomes More Challenging

Cultural messages related to sexual relations are many, frequently contradictory, and variable in time and setting. We divided the theme of experiencing sexuality as narrated by participants into four sub-themes.

#### Lowered Trust in Self and Others

At different stages of the illness, patients can be aware that their behavior could be changed. Sometimes, though not frequently, this involves sexuality. Witnessing other patients’ behaviors in the psychiatric ward can make trusting oneself and others more difficult.

Martha tells a story about episodes of brief sexual encounters with various men during her illness:

*You know, I was seeing these men, who only wanted sex from me and nothing else (…) I don’t know if it was the illness, or if it was my … my sexual awakening. I was just going around … going around…trying to pick up guys for sex. Some agreed, some didn’t. You know how it goes… sometimes I’d be at the man’s house, and in the morning I’d leave to take care of my things. And with some the sex happened in the car.* Interviewer: *You mentioned it used to happen, meaning it doesn’t happen anymore?* M: *Because I started taking medication.*

Martha’s full story is that of a young woman fondly recalling romantic love before the onset of her illness, and then describing various sometimes frightening experiences during her illness, and accepting her current lowered libido. It introduces the next theme, characteristic for most of the female participants. It deals with how the patients perceive the risk of sexual abuse of an ill woman by men.

#### Women’s Experiences: Risk of Sexual Abuse and Need for Stable Relationships

Three out of five of the female participants described situations which they eventually came to consider as abuse. Each of them desired love and pursued a long-term close relationship but experienced rather short-term relations based primarily on sexuality. What is striking in these narrations is the disproportionality of the women’s dreams and desires versus how their relations with men happened in reality, as well as the imbalance of power in the relationships. For example, the imbalance pertained to age—where a man was much older, and the woman was teenage and ill, or with a difference in health status and ability to think critically.

This is how Emily describes her submissiveness toward a mentally healthy and much older partner, with whom she was in love at one point: *“He wanted … I mean he abused me sexually. I felt that he’s abusing me. (…) I was defending myself from the attacks of his bad behavior”.*

Despite this, it took her a long time before she terminated this relationship; it was difficult for Emily to trust her feelings and to determine if the relationship she was in was loving or abusive. Her family and the people around her had no doubts that she was being abused, but her own inner experience is colored by confusion, by the hope for love, and by the bitter realization that she was offered only a much less meaningful experience. Such experiences can discourage sexuality in any form.

Afterward, I thought to myself if I were to find another man, it would be a “white marriage,” you know, a relationship with no sex life. I was really determined to… I’ve been abstinent for… a year, year and a half, yeah. But back then I was just thinking that I don’t want to have any interactions with any men.

In various situations, the female participants referred to their own sexual needs, which varied throughout different periods of their lives, sometimes clear and strong, sometimes suppressed by medication. Illness made it difficult to satisfy those needs in a way that would match their desires, fantasies, or values.

#### Men’s Experiences: Romantic Ideals and Competition

While women’s narratives on sexuality were complex, elaborate, and varied, men’s narratives were shorter and less open. It is important to note that the interview conditions were not equivalent for the male and female participants because all interviews were conducted by a young female interviewer. This could act as a limitation in the case of male participants, but an advantage for female participants, who may in turn feel safer while expressing their sexual experiences. In the interviews with male participants, the motif of sexuality was narratively linked with awareness of their low social status. This evoked the theme of competition with other men. Participants also spoke about difficulties in managing jealousy.

For example, Jack says that his 2-week-long love “vanished” and that he would rather “it better not come back.” For him, love is associated with risks and aggression, both expressed and experienced. When the object of his affection chose another man—also a patient—at a club, Jack reacted with aggression toward both of them, for which he was thrown out of the club: “*Yeah, she went to fuck another guy. She was dating me. So, being the clever guy I am, I whooped her ass and bid her good night. So they threw me out of the club. Banned me for 3 months.”*

His thoughts about a future relationship are colored by suspicions that the partner would cheat on him. Therefore, he sums it all up with the saying: “*Love is a feeling that’s dumb, it ends in tears, it ends on your bum”.*

The brutality of the rivalry for sexual relations with women in the narratives of longer-suffering respondents stands in contrast to the stories of more-recently diagnosed participants. The latter focus on their ideals—long-term, harmonious, loving relationships with women. Those are the relationships which they seek and dream of, despite having experienced plenty of rejection. For them, love is an ideal, but the naked reality they experience can be unforgiving.

#### Voluntary Sexual Abstinence

Another theme which appeared in the narrations concerning sexuality was that of voluntary sexual abstinence, frequently associated with giving up, entirely or temporarily, on the search for new relationships. One of the reasons is medication, whose side effects include libido suppression. Alice:

The meds suppress libido and so I, for example, right now I don’t have this sort of… how do I call it… I don’t have situations where I would feel sexual attraction to a man, that I would have a physical response to someone, I’m just dampened in that respect.

For some of the participants, sexuality is very important, and yet they choose abstinence, despite having sexual needs. For a few of the participants, the choice was motivated by religious beliefs. They did not want to have casual or extramarital relations, and the other kinds were not available to them; and in their solitude, religion was an important source of support. Such motifs appeared in the narrations of three of the five female participants. Women also spoke about the need for abstinence arising from disillusionment with their past experiences and the excessively high perceived risk associated with this aspect of life.

### Theme 5—Ways of Coping

#### Love Is Understood as a Universal Value

Both the need for love and the difficulties in finding it and building a lasting relationship are seen by our patients as universal experiences, and not just through the lens of their illness; on the contrary, they are seen as part of the human condition.

Bella has been ill for 4 years and is now in her forties. She is divorced and recalls that many of her healthy friends back from when they were schoolmates are now divorced, as well. She believes that the fact that love does not work out is just the human condition. Not only the ill are lonely; for her, loneliness and rejection are a part of destiny, especially women’s destiny. When an ill person faces loneliness or abandonment, her or his fate is shared with many people before them, including healthy people.

Most participants’ narratives reveal a strong belief that love is one of the most important values in—and gives meaning to—life. However, romantic and erotic love is viewed neither as the only nor as the most important kind of love. Several participants point out spiritual values. Eva names agape her ideal of love: *“Agape love is the most perfect kind of love (…) Love is … indeed one of the most important values in life, which everybody pursues, which we all are searching for, without which we can’t live, we can’t be happy.”* Agape love, seen somewhat in opposition to erotic love, is her ideal, not only in the context of male–female relationships but also in the context of friendship, and it gives meaning to her life. The need for honesty, dedication, and commitment appears as an important motif in the participants’ narratives.

#### Other Objects of Love

Many of the participants talk about objects of love, who—as they put it—“substitute for” or “fill the gap” caused by the lack of a partner, and who may fulfill the patients’ needs frustrated due to the lack a romantic partner. For some of them, that person is their mother. For example, Jack, when asked what love is the most important to him, answers without a second thought: “*love for my mother.”* Emily gives a long list of people she loves, starting with her elderly mother—whose death she fears and dreads—going on to other family members, mentioning her sister’s children, as well as persons met at various meetings and support groups, and finally ending with God. For her, helping others and self-development are also forms of love.

These types of responses are common—relations with objects of love, parents, relatives, friends, and in some cases, God, are an opportunity to give and to express love, but are also mutual and can satisfy the patients’ basic needs. Alice:

I mean, for me, I think I can say that my mom is my substitute for a relationship. I can’t imagine functioning without other people and I think that if I didn’t have my mom, I would really need someone to be with me. But also the illness has affected that – we’re now closer to one another, we have more tolerance, we talk more, we spend more time together. (…)It’s a sort of emotional or psychological hunger. To some extent, you can get that from a person, with whom you’re not necessarily in a relationship.

The clear presence of religious coping in four of the ten narratives is noteworthy. Spiritual needs are expressed, and a relationship with God was identified explicitly as important in this regard. Martha: *“There are no men. I believe that God is my man. It’s the illness, I know.”* Even though Martha self-stigmatizes her confession that God serves the role of her life partner, the cultural notion of closeness to God as something that can fulfill an individual’s emotional and inner needs is a valid one, also within the Catholic religion to which she confesses.

#### Loneliness

Despite the above, the participants still experience loneliness. James:

I think that (managing life) would be easier with someone. It’s definitely easier to deal with any problem if you have someone to help you. (…) Maybe I’ve gotten a little bit used to loneliness. I mean not that sort of painful, piercing loneliness, even though it’s like that sometimes. There are times when I feel that kind of loneliness. I would definitely prefer it with someone, but time will tell.

#### Expectations for the Future

The participants’ expectations for their futures varied widely. John is making an active effort to find love: *“Now I’m working on…on…on relations with women. That’s what I’m focused on, to be honest”.*

Harry is also actively working on self-improvement. He has achieved a measure of success in physical fitness—for some time now, he has been jogging regularly (to lose weight and stay in shape)—and he also wants to work on his financial stability to be able to use these as a basis for a long-term relationship. Oliver also has hopes for the future and wishes to find a stable relationship. In contrast, Alice believes it is possible to maintain lasting and close relationships full of love and acceptance only if they existed before the onset of the illness, but she is critical of relationships initiated during illness. She believes that she has changed inside, so much that she will probably never be personally ready for love.

Yeah, yeah… I mean that pessimistically. I think it would be more pessimistic if I felt that, at the present time, I was ready for those things but couldn’t carry them out. But I just feel that I’m not ready, and I can’t imagine being ready. So, it’s a little easier for me this way.

In general, the narratives of younger patients with a shorter history of illness expressed more hope for future relationships, while longer-suffering patients were less optimistic in their expectations.

## Discussion

The common theme for persons in this study in the context of psychosis and love relationships was the need to adapt to the illness. Illness onset was often a turning point for their previous relationships and challenged the identities which they had before the onset of illness. Illness onset seems to be a time that puts a special strain on interpersonal relationships, including love relationships. There was a need to deal with the stigma associated with being an in-patient at a psychiatric hospital, as this stigma sometimes becomes a personal identity label (Corrigan and Watson, 2002). This result is consistent with quantitative research done on the influence of self-stigmatization (Brohan et al., 2010). There is a vicious cycle, in which the self-stigmatization leads to (self-)isolation, which decreases social functioning, which in turn amplifies the stigma. Thus, from an interactive social point of view, stigma becomes a determinant of the illness, not just its consequence.

Stigmatization processes, and especially the harmful impact of institutionalization, were subject to very lively debate within the anti-psychiatry movement (Goffman, 1968; Burns and Foot, 2020). Authors within this tradition stress the role of institutionalized psychiatry in producing the very stigma of mental illness, rather than working against it (Burns and Foot, 2020). This debate was also present in the post-communist countries, although for historical reasons later and less intensely than in Western Europe; thus, it could not be experienced to the same extent as in Western Europe (Moskalewicz et al., 2020). The legacies of communism involve large over-institutionalization in psychiatric healthcare and underdeveloped services of primary care, factors that are structural and slow to change (Jenkins et al., 2005). The present study was conducted in Poland, a central European post-communist country, where these problems, especially psychiatric healthcare underfinancing, are still persistent. Advances in promoting more wholesome, less institutionalized psychiatric care are much debated, but in practice the progress is slow. As demonstrated in the present study, this has serious consequences for those affected (Świtaj et al., 2012).

Love relationships were perceived by participants as made more complicated by internal obstacles caused by their illness. What is described in the literature as deficits in emotional, cognitive, and social skills (Penn et al., 2008; Hooley, 2010) from a phenomenological perspective was described by patients as losing a part of themselves, change in emotions, and difficulties in integrating different beliefs. They reported diminishing degrees of trust toward self and others, as well as toward their own phenomenological modalities: their own memories, feelings, perceptions, and desires. However, regardless of the chaos and residual impacts of psychotic experiences that sometimes persist over the longer term, all participants were able to express nuanced feelings and attitudes toward love—through either reflection, grievance, longing, anger, or irony. The amount of work and effort put into assimilating, communicating, and making sense of the illness and incongruent psychotic experiences, which was evident throughout the interviews, commands respect. It also makes clear the value of psychotherapy or psychological assistance in those situations. Several participants in our study took advantage of psychotherapy, psychological counseling, and/or peer support groups, and they valued the possibility to talk about their relationship experiences in this context.

External difficulties were another common feature for the participants’ experiences. The discrimination and stigma of mental illness caused most of them to experience rejection in relationships. The significance of poverty concerning practical possibilities of love and romance was striking and grew with time, being the strongest factor in persons who have been ill for an extended time. This corresponds to the conclusions from population data (Cohen, 1993; Saraceno and Barbui, 1997), which demonstrates that poverty is a risk factor for negative outcomes and prognosis of mental illness. Our study points to one of the possible explanatory mechanisms here, via impoverishing one’s social relationships.

Sexuality is one of the areas where many unmet needs are reported by the patients, when it comes to treatment and psychiatric care (Volman and Landeen, 2007). This is additionally important because sexual dysfunction is among the reasons for non-compliance with antipsychotic medication (Kelly and Conley, 2004). The participants in our study agreed that sexuality becomes riskier in their situations, and the risks of rejection, abuse, and violence in this context were perceived as high. An especially worrying factor was the high risk of sexual abuse reported by women in our study. Studies show a larger prevalence of sexual trauma among women with schizophrenia (Darves-Bornoz et al., 1995), and the risk that their experiences in this regard can be unseen and unreported (Rice, 2008). On the other side, the basic agency and freedom in choosing one’s own expressions of sexuality should not be limited by overprotective attitudes of mental health professionals (Urry and Chur-Hansen, 2018). From an experiential perspective, sexual trauma can change the meanings ascribed to sexuality in general, especially causing one to reject it, or to resign from entering sexual experiences with other persons. This trajectory of changing attitudes was visible in women’s narratives in our study. Moreover, sexual abuse can lead to mistrust or disillusionment, which constitutes a barrier for entering romantic relationships.

All participants expressed needs related to love, but some also spoke about a lack of readiness or hope for a romantic relationship, especially if it were to be sexual as well. The stories of various difficulties are also a subtle portrayal of the strategies for overcoming them. This can be seen, among other forms, in the ways in which our participants communicated: it features humor, pensiveness, altruism, distance, spirituality, sensitivity, understanding, and acceptance. Love as a need to give of oneself was expressed through helping others, especially other chronically ill individuals through support groups. Even individuals whose illness severely limited them spoke about the need to give of themselves, if only through physical labor, or contributing to the public good in other ways, however, small. Davidson (2011) writes in a similar vein about the restorative power of non-romantic love types, such as *philia*, a mutual love between friends and family, or within a community, and *thelema*, the sense of contributing to society or a group. Love understood as a need to be loved was also fulfilled in various contexts, such as through family relationships, friendships, and spirituality. Relations with the opposite sex were characterized by ideals, which sometimes were contrary to life experiences, especially the ideal of a long-lasting and stable relationship, which was present in both men and women.

Thinking about psychiatric healthcare in terms of recovery-oriented practice (Davidson, 2011) calls for special awareness to love, as one of the central aspects of life and of personhood. It is the very sense of personhood that can be threatened by psychotic experiences. The nature of love experiences makes being a person salient, rather than focusing on symptoms and diagnosis. Here lies the value of including the area of love in recovery-oriented practices within and outside healthcare.

To sum up: the understanding of love within our group of participants shares features that are universal for human experiences of love. This applies to the expression of the need for love in several dimensions—love as an abstract, to love, and to be loved, which matters significantly, and can be a decisive factor in determining the course of one’s life. Also, cultural values and expectations are usually shared, most notably the value of stable loving relationships, which last over a lifetime and provide mutual support and are rich, genuine feelings for each other. The understanding of love also shares some features with other group experiences, such as those who endure long-term illness, poverty, or discrimination and stigmatization. These experiences are expressed in a shared vulnerability, which makes love more difficult to achieve, but also for the same reason more valued and nuanced in its expression, as effort is required to maintain and grow relationships against external hardship. The very experience of psychosis adds another layer to these common stories of the struggle for love. It taps deeply into the sense of person’s identity, thus risking self-stigma and renouncement or avoidance of relationships. It shakes the sense of trust toward one’s own judgment, own thoughts, desires, and sexuality, as experienced by the participants of our study. It also sometimes produces distinct, intense experiences, such as hearing voices. Voice personalization adds specific meaning to love and intimacy, but at the same time it can practically exclude others from the inner experiential world of a person, thus deepening interpersonal isolation (McCarthy-Jones and Davidson, 2013). In the case of our participants, it was not one but many of these processes simultaneously, both from within and from external circumstances that colored their experiences and understandings of love. At the same time, each person’s story and attitude remains unique.

### Limitations

The nature of the IPA method is idiographic. Our results, even if they mirror to some extent certain common difficulties of patients, cannot be generalized. Moreover, we are aware of the extreme diversity of psychotic experiences, even within the formal diagnosis of schizophrenia, thus making “psychosis spectrum” perhaps a better term. This diversity makes some researchers doubt if the schizophrenia group can, in fact, be seen as one diagnosis, or rather many (Guloksuz and van Os, 2018). Our study captures only a part of the possible spectrum of the illness, and many prevalent forms of it are not represented. The same can be said about the diversity of love experiences; some of them are also not present in our sample, for example, long-term stable married couples, or LGBTQ experiences. Participants’ living conditions, duration of illness, and occurrence of first episodes varied in our study, which is also a limitation, as a more homogenous sample would be preferable in IPA methodology.

Engaging in self-reflexivity also made us aware of some further limitations and interpretational boundaries. All of the authors are psychologists and have some psychotherapeutic backgrounds. Thus, our perspectives may be different from that of other mental health professionals. We also have experience with psychotherapeutic work with psychotic patients and with accompanying psychiatric support groups for persons with long-term mental illness. Thus, this study explicitly intends to show the difficulties and emotions of this group of patients in a way that would give justice to those experiences in all of their complexity and fight the stigma. For that reason, we may be less focused in our analysis on deficits sometimes brought on by illness, especially when it comes to negative symptoms, such as severely impoverished affect and passivity. Even if we did not purposefully influence the sampling of participants, patients with predominantly negative symptoms were less likely to be in our sample. Taking part in an IPA study requires personal motivation on the part of the patient, openness and willingness to engage in an in-depth conversation, and a willingness to give testimony to one’s own experiences.

## Data Availability Statement

The datasets generated for this study are not readily available because “This is a qualitative study based on the interviews, the interviews are not available as full data, as it would threaten the participant’s anonymity”. Requests to access the datasets should be directed to magda.budziszewska@gmail.com.

## Ethics Statement

The studies involving human participants were reviewed and approved by Board for Research Ethics – Faculty of Psychology, University of Warsaw. Written informed consent for participation was not required for this study in accordance with the national legislation and the institutional requirements. However, the ethics committee approved the protocol of receiving oral informed consent.

## Author Contributions

MDB: planning the study, designing methods, designing interview protocol, initial analysis, collaborating in the analysis and refining it, and writing the major part of the manuscript. KW: designing interview protocol, conducting the interviews, collaborating in the analysis and refining it, and reviewing the written manuscript. MB-H: collaborating in the analysis and refining it, writing minor parts of the manuscript, reviewing the written manuscript, and editing. All authors contributed to the article and approved the submitted version.

## Conflict of Interest

The authors declare that the research was conducted in the absence of any commercial or financial relationships that could be construed as a potential conflict of interest.
